# Genome-wide characterization of simple sequence repeats in Palmae genomes

**DOI:** 10.1007/s13258-020-00924-w

**Published:** 2020-04-03

**Authors:** Manee M. Manee, Badr M. Al-Shomrani, Mohamed B. Al-Fageeh

**Affiliations:** 1 National Center for Biotechnology, King Abdulaziz City for Science and Technology, Riyadh, Saudi Arabia; 2 Center of Excellence for Genomics, King Abdulaziz City for Science and Technology, Riyadh, Saudi Arabia; 3 Institute of Bioinformatics, University of Georgia, Athens, GA USA

**Keywords:** Arecaceae, Palmae family, Microsatellite, SSR abundance, Molecular marker

## Abstract

**Background:**

Microsatellites or simple sequence repeats (SSRs) have become the most significant DNA marker technology used in genetic research. The availability of complete draft genomes for a number of Palmae species has made it possible to perform genome-wide analysis of SSRs in these species. Palm trees are tropical and subtropical plants with agricultural and economic importance due to the nutritional value of their fruit cultivars.

**Objective:**

This is the first comprehensive study examining and comparing microsatellites in completely-sequenced draft genomes of Palmae species.

**Methods:**

We identified and compared perfect SSRs with 1–6 bp nucleotide motifs to characterize microsatellites in Palmae species using PERF v0.2.5. We analyzed their relative abundance, relative density, and GC content in five palm species: *Phoenix dactylifera*, *Cocos nucifera*, *Calamus simplicifolius*, *Elaeis oleifera*, and *Elaeis guineensis*.

**Results:**

A total of 118241, 328189, 450753, 176608, and 70694 SSRs were identified, respectively. The six repeat types were not evenly distributed across the five genomes. Mono- and dinucleotide SSRs were the most abundant, and GC content was highest in tri- and hexanucleotide SSRs.

**Conclusion:**

We envisage that this analysis would further substantiate more in-depth computational, biochemical, and molecular studies on the roles SSRs may play in the genome organization of the palm species. The current study contributes a detailed characterization of simple sequence repeats in palm genomes.

**Electronic supplementary material:**

The online version of this article (10.1007/s13258-020-00924-w) contains supplementary material, which is available to authorized users.

## Introduction

Plants in the palm family (Arecaceae or Palmae) are important economic crops that are widely cultivated in arid and semi-arid regions of North Africa, the Sahara, the Middle East, and eastward to the Indus Valley. Palmae is a distinct family of monocotyledon species with up to 2800 species currently known, which are distributed over 202 genera (Xiao et al. 2016). Palm plants are critical ecological and socioeconomic resources for many countries, including Saudi Arabia; they play important roles in food security, wood for building, ornamentals, and industrial materials (Barrow 1998; Aberlenc-Bertossi et al. 2014). The date palm (*Phoenix dactylifera*), coconut (*Cocos nucifera*), and African oil palm (*Elaeis oleifera*) are the most economically important fruit crops in the palm family. There are more than 3000 cultivars of date palm worldwide, of which 60 are considered to be important in the global market (Moussouni et al. 2017). Despite the increasing number of genomic studies on Palmae trees, little genome-wide characterization has been performed on these plants for the purposes of conservation and genetic assessment.

Assessment of genetic diversity is crucial for the conservation of palm cultivar germplasm. Estimates of the genetic diversity of palm plant germplasm have traditionally been based on morphological information (Elhoumaizi et al. 2002). However, morphological markers do not reliably provide accurate assessments because they are highly affected by environmental factors. Molecular markers are more informative at any developmental stage of the plant. Molecular breeding through marker-assisted selection would also expedite the genetic improvement of palm cultivars (Zhao et al. 2012). Microsatellites or simple sequence repeats (SSRs) are very useful markers for the analysis of plant diversity. In addition, SSR markers can be used for DNA fingerprinting to distinguish among closely-related palm cultivars. SSRs are tandem repeats of one to six base pairs per repeat unit, and are widely distributed in eukaryotic and prokaryotic genomes (Xu et al. 2016; Yang et al. 2003). Rapid expansions and contractions of these repeats due to replication slippage may make them useful for carrying out population genetics studies within a species (Huntley and Golding 2006).

The recent release of draft whole-genome sequences for several palm species provides an opportunity to carry out post-genomic analysis in order to identify and compare the distributions of SSRs across palm genomes. To date, draft genome sequences have been released for five species in the Palmae family: *P. dactylifera*, *C. nucifera*, *Calamus simplicifolius*, *E. oleifera*, and *E. guineensis*. This study aimed to screen these five genome sequences for microsatellites, detect SSR motifs, and analyze the frequency and distribution of SSRs.

## Materials and methods

### Genome sequences

Draft genome sequences for five palm tree species (*P. dactylifera*, *C. nucifera*, *C. simplicifolius*, *E. oleifera*, and *E. guineensis*) were selected for the analysis of SSR distributions at the genomic level. These genomes have been assembled at the scaffold level according to the genomic resources of the National Center for Biotechnology Information (NCBI). The genome sequences with accession numbers of GCA_000413155.1, GCA_003604295.1, GCA_900491605.1, GCA_000441515.1, and GCA_001672495.1 were downloaded in FASTA format from the Genomes FTP site (ftp://ftp.ncbi.nlm.nih.gov/genomes/).

### Genomic quality assessment

Completeness of the genome assemblies was assessed with Benchmarking Universal Single Copy Ortholog (BUSCO) v3.0.2 (Simão et al. 2015) with default settings. BUSCO genes are good candidates for evaluating genome completeness because from an evolutionary perspective, they are expected to be found in the tested genome (Simão et al. 2015; Waterhouse et al. 2017). The BUSCO tool analyzed each genome assembly state in terms of complete BUSCOs, complete and single-copy BUSCOs, complete and duplicated BUSCOs, fragmented BUSCO, and missing BUSCOs using a plant-specific database (embryophyta_odb9) that consisted of 1440 total BUSCO groups from 30 species.

### Identification of microsatellites

Genome-wide SSR mining was performed by scanning each entire genome with the software PERF v0.2.5 (Avvaru et al. 2017). A number of criteria were adopted to identify perfect SSRs. Specifically, repeat sizes of 1 to 6 nucleotides long were searched, and minimum repeat numbers were restricted to 12 repeats for mononucleotides, 7 repeats for dinucleotides, 5 repeats for trinucleotides, and 4 repeats for tetra-, penta- and hexanucleotides, consistent with previous studies (Liu et al. 2017; Qi et al. 2018). The remaining parameters were set as default. Repeats with unit patterns being circular permutations and/or reverse complements were deemed as one type in this study (Jurka and Pethiyagoda 1995; Li et al. 2009). For instance, ACT contains ACT, TAC, CTA, TGA, ATG, and GAT in different reading frames or on the complementary strand. Different types of SSR repeats or motifs were compared in terms of relative frequency (the number of SSRs per Mb) and relative density (the total length of SSRs in bp per Mb). All graphical and statistical analyses were performed in the R programming environment (version 3.4.3) (R Core Team, 2017).

## Results

### Assessing the completeness of the genome assemblies

**Fig. 1:**
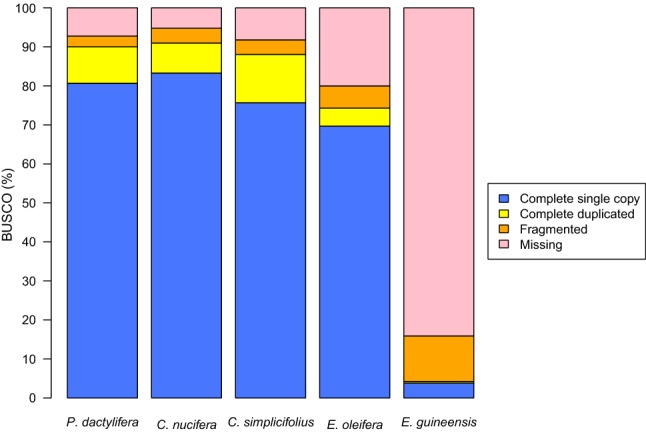
Genome assembly evaluation. The BUSCO embryophyta_odb9 dataset, including 1440 BUSCOs, was used to assess the five genome assemblies

We adopted the BUSCO plant lineage dataset, which consisted of 1440 single-copy orthologs for the Embryophyta lineage, to assess the completeness of each of the five genome assemblies. The *C. nucifera* genome assembly had the highest BUSCO scores among those surveyed (Fig. 1), with 1311 (91%) complete BUSCOs (1200 complete single-copy and 111 complete duplicated BUSCOs); 3.80% of sequences were fragmented (54 BUSCOs) and 5.20% were considered missing (75 BUSCOs). The BUSCO scores of *C. nucifera*, *P. dactylifera*, and *C. simplicifolius* genome assemblies were comparable, and higher than the two palm assemblies from genus *Elaeis* (*E. oleifera* and *E. guineensis*). However, the *E. guineensis* genome assembly showed low BUSCO scores relative to all four of the other assemblies (Fig. 1). In the *E. guineensis* genome assembly, only 60 (4.20%) complete BUSCOs were identified (54 complete single-copy and 6 complete duplicated BUSCOs).

### Identification and characterization of microsatellites in palm genomes

In the five palm genomes (*P. dactylifera*, *C. nucifera*, *C. simplicifolius*, *E. oleifera*, and *E. guineensis*), a total of 118241, 328189, 450753, 176608 and 70694 perfect SSRs were identified (Files S1–S5) with frequencies ranging from 125.90 to 229.88 SSR/Mb, respectively (Table 1). About 0.40, 0.36, 0.44, 0.23, and 0.26% of the genome was occupied by perfect SSRs, respectively. The relative densities ranged 2297.25–4373.04 SSR/Mb, while the mean lengths of SSRs were approximately 19 bp for *C. nucifera* and *C. simplicifolius*, and approximately 18 bp for *P. dactylifera*, *E. oleifera*, and *E. guineensis*.

**Table 1:** Overview of the five palm genomes

Parameter	*P. dactylifera*	*C. nucifera*	*C. simplicifolius*	*E. oleifera*	*E. guineensis*
Common name	Date palm	Coconut palm	Rattan palm	American oil palm	African oil palm
Genome size (Mb)	556.48	1839.17	1960.81	1402.73	499.03
GC content (%)	35.86	31.79	39.65	28.12	32.10
Number of SSRs	118241	328189	450753	176608	70694
Total length of SSRs (bp)	2233944	6522297	8574690	3222415	1279910
Average length (bp)	18.89	19.87	19.02	18.25	18.11
Frequency (SSR/Mb)	212.48	178.44	229.88	125.90	141.66
Density (bp/Mb)	4014.41	3546.32	4373.04	2297.25	2564.80
Genome SSRs content (%)	0.40	0.36	0.44	0.23	0.26

**Table 2:** Number, length, frequency, and density of mono- to hexanucleotide repeats in palm genomes

Repeat type	Parameter	*P. dactylifera*	*C. nucifera*	*C. simplicifolius*	*E. oleifera*	*E. guineensis*
Mono-	Number of SSRs	35942	142586	218192	53939	20684
	Total length (bp)	485527	2502957	3293664	743482	282341
	Average length (bp)	13.51	17.55	15.10	13.78	13.65
	Frequency (SSR/Mb)	64.59	77.53	111.28	38.45	41.45
	Density (bp/Mb)	872.50	1360.92	1679.75	530.03	565.78
Di-	Number of SSRs	46830	96689	139482	74362	28630
	Total length (bp)	1058068	2245158	3490730	1556956	567482
	Average length (bp)	22.59	23.22	25.03	20.94	19.82
	Frequency (SSR/Mb)	84.15	52.57	71.14	53.01	57.37
	Density (bp/Mb)	1901.36	1220.75	1780.25	1109.95	1137.17
Tri-	Number of SSRs	18603	33229	47311	22446	6616
	Total length (bp)	350310	619926	885591	415284	127803
	Average length (bp)	18.83	18.66	18.72	18.50	19.32
	Frequency (SSR/Mb)	33.43	18.07	24.13	16.00	13.26
	Density (bp/Mb)	629.51	337.07	451.65	296.06	256.10
Tetra-	Number of SSRs	12396	33721	30357	19309	11425
	Total length (bp)	236976	662892	556244	356136	224040
	Average length (bp)	19.12	19.66	18.32	18.44	19.61
	Frequency (SSR/Mb)	22.28	18.34	15.48	13.77	22.89
	Density (bp/Mb)	425.85	360.43	283.68	253.89	448.95
Penta-	Number of SSRs	3149	18699	11011	4762	2402
	Total length (bp)	68845	408660	233375	102875	52750
	Average length (bp)	21.86	21.86	21.20	21.60	21.96
	Frequency (SSR/Mb)	5.66	10.17	5.62	3.40	4.81
	Density (bp/Mb)	123.72	222.20	119.02	73.34	105.71
Hexa-	Number of SSRs	1321	3265	4400	1790	937
	Total length (bp)	34218	82704	115086	47682	25494
	Average length (bp)	25.90	25.33	26.16	26.64	27.21
	Frequency (SSR/Mb)	2.37	1.78	2.24	1.28	1.88
	Density (bp/Mb)	61.49	44.97	58.69	33.99	51.09

**Fig. 2:**
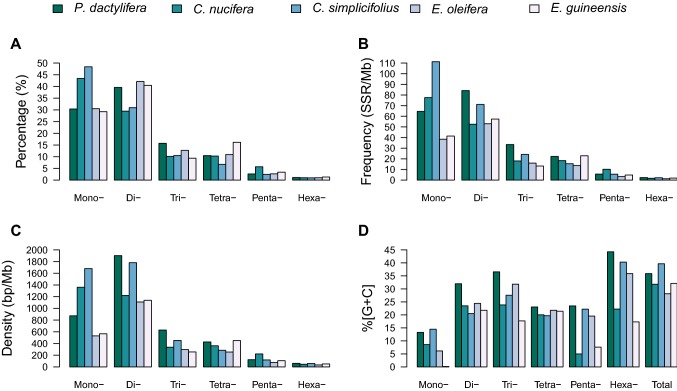
Comparison of percentage, frequency, density, and GC content of SSRs in palm genomes. Percentages were calculated according to the total number of each SSR type divided by the total number of SSRs. ABCD represent percentage, frequency, density, and GC content of SSRs, respectively

The number, length, relative frequency, relative density, and percentage of the six types of SSRs are shown in Table 2. The percentage, relative frequencies, and densities of different SSR types were found to vary greatly between the five palm genomes (Fig. 2). Dinucleotide SSRs were the most frequent type in *P. dactylifera*, *E. oleifera*, and *E. guineensis*, with the highest frequencies of 84.15, 53.01, and 57.37 SSR/Mb, accounting for 39.61, 42.11, and 40.50% of SSRs in these genomes, respectively (Fig. 2a, b). Mononucleotide SSRs were the most abundant type in *C. nucifera* and *C. simplicifolius*, with the highest frequencies of 77.53 and 111.28 SSR/Mb, occupying about 43.45 and 48.41% of all SSRs in those genomes, respectively. Mononucleotide SSRs were also the second most frequent in *P. dactylifera*, *E. oleifera*, and *E. guineensis*, while dinucleotide SSRs were the second most abundant type in *C. nucifera* and *C. simplicifolius*. Tri- and tetranucleotide SSRs were more frequent than pentanucleotide SSRs in all five genomes. Hexanucleotide SSRs were the least abundant across all five genomes, with a frequency of below 2.38 SSR/Mb, and accounted for only 1.12, 1.00, 0.98, 1.01, and 0.07% of all SSRs in these genomes, respectively (Fig. 2b). Dinucleotide SSRs were found to have the highest densities, ranging from 1109.95 to 1901.36 bp/Mb in *P. dactylifera*, *C. simplicifolius*, *E. oleifera*, and *E. guineensis*, whereas mononucleotide SSRs had the highest density (1360.92 bp/Mb) in *C. nucifera* (Fig. 2c).

The GC content of different types of SSRs was investigated for the five genomes (Fig. 2d). Hexanucleotide SSRs had the highest GC content in *P. dactylifera* (44.24%), *C. simplicifolius* (40.28%), and *E. oleifera* (35.87%). Trinucleotide SSRs were found to have the highest GC content in *C. nucifera* (23.83%), while dinucleotide SSRs had the highest GC content in *E. guineensis* (21.76%). Mononucleotide SSRs were found to have the least GC content in *P. dactylifera*, *C. simplicifolius*, *E. oleifera*, and *E. guineensis*, at only 13.29, 14.50, 6.12, and 0.16%, respectively, while pentanucleotide SSRs had the least GC content in *C. nucifera*, at only 4.97%.

### Abundance and repeat numbers for different microsatellite motifs

The microsatellites in palm genomes were determined to be relatively AT-rich. To gain insight into this characteristic, we analyzed SSR motif composition. The most abundant SSR motifs were found to vary with species. The degenerated number of repeat motifs was found to be 2, 4, and 10; these were identical between species for mono- to trinucleotide repeat types and were different for tetranucleotide, pentanucleotide, and hexanucleotide repeat types.

#### Mononucleotide repeats

**Fig. 3:**
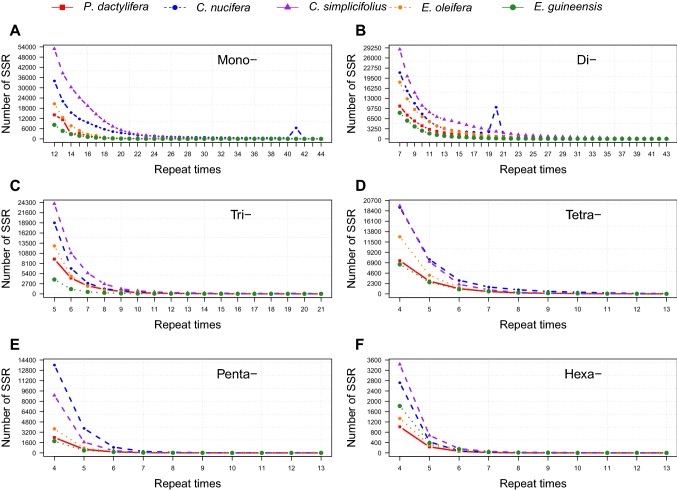
Repeat counts for different SSR types in the palm genomes. ABCDEF represent mono-, di-, tri-, tetra-, penta-, and hexanucleotide SSR types, respectively

The predominant mononucleotide motif type was $(\hbox {A})_{\mathrm{n}}$, with a total number of 31368, 131905, 188454, 50637 and 20651 SSRs in the five genomes, accounting for 87.27, 92.51, 86.37, 93.88, and 99.84% of all mononucleotide SSRs, respectively (Table 3). The frequencies and densities of $(\hbox {A})_{\mathrm{n}}$ were 36.10–96.11 SSR/Mb and 497.57–1436.24 bp/Mb, respectively, while the average lengths were 13.42–17.35 bp. The $(\hbox {C})_{\mathrm{n}}$ motif type was far less abundant than $(\hbox {A})_{\mathrm{n}}$, accounting for only 0.16–13.63% of all mononucleotide SSRs in the five genomes. Mononucleotide repeats ranged from 12 to 115, 12 to 44, 12 to 175, 12 to 65, and 12 to 83 repeats in length, respectively. Repeat numbers between 12–22, 12–41, 12–36, 12–24, and 12–21 accounted for 99.18, 99.98, 99.71, 99.01, and 98.66% of the total number of mononucleotide SSRs in these genomes, respectively (Fig. 3a).

**Table 3:** Number, length, frequency, and density of the most frequent SSR motifs for each SSR type in palm genomes

Repeat motif type	Parameter	*P. dactylifera*	*C. nucifera*	*C. simplicifolius*	*E. oleifera*	*E. guineensis*
A	Number of SSRs	31368	131905	188454	50637	20651
	Total length (bp)	421010	2288571	2816200	697952	281897
	Frequency (SSR/Mb)	56.37	71.72	96.11	36.10	41.38
	Density (bp/Mb)	756.56	1244.35	1436.24	497.57	564.89
C	Number of SSRs	4574	10681	29738	3302	33
	Total length (bp)	64517	214386	477464	45530	444
	Frequency (SSR/Mb)	8.22	5.81	15.17	2.35	0.07
	Density (bp/Mb)	115.94	116.57	243.50	32.46	0.89
AT	Number of SSRs	16405	49505	73131	36840	15913
	Total length (bp)	384960	1194804	2064214	801040	321888
	Frequency (SSR/Mb)	29.48	26.92	37.30	26.26	31.89
	Density (bp/Mb)	691.78	649.64	1052.74	571.06	645.03
AG	Number of SSRs	24498	37493	56621	27114	6043
	Total length (bp)	548096	854432	1214612	549626	115996
	Frequency (SSR/Mb)	44.02	20.39	28.88	19.33	12.11
	Density (bp/Mb)	984.93	464.57	619.44	391.83	232.44
AC	Number of SSRs	5716	9393	9444	10139	6583
	Total length (bp)	121652	191274	207532	202016	128190
	Frequency (SSR/Mb)	10.27	5.11	4.82	7.23	13.19
	Density (bp/Mb)	218.609	104.00	105.84	144.02	256.88
AAT	Number of SSRs	4702	14147	15355	6666	3073
	Total length (bp)	100233	287169	324243	137616	66840
	Frequency (SSR/Mb)	8.45	7.69	7.83	4.75	6.16
	Density (bp/Mb)	180.12	156.14	165.36	98.11	133.94
AAG	Number of SSRs	5349	10634	14152	7636	2179
	Total length (bp)	97662	187221	245298	135789	37794
	Frequency (SSR/Mb)	9.61	5.78	7.22	5.44	4.37
	Density (bp/Mb)	175.50	101.80	125.10	96.80	75.74
AGG	Number of SSRs	2977	2357	3512	2774	204
	Total length (bp)	54216	41088	63447	48822	3342
	Frequency (SSR/Mb)	5.35	1.28	1.79	1.98	0.41
	Density (bp/Mb)	97.43	22.34	32.36	34.81	6.70
ATC	Number of SSRs	1501	1846	7215	1301	623
	Total length (bp)	25269	31590	130437	22332	10743
	Frequency (SSR/Mb)	2.70	1.00	3.68	0.93	1.25
	Density (bp/Mb)	45.41	17.18	66.52	15.92	21.53
ACAT	Number of SSRs	2598	11404	4124	6140	6786
	Total length (bp)	54624	260300	85240	123208	141664
	Frequency (SSR/Mb)	4.67	6.20	2.10	4.38	13.60
	Density (bp/Mb)	98.16	141.53	43.47	87.84	283.88
AAAT	Number of SSRs	4217	11376	10768	5458	2406
	Total length (bp)	79320	205164	192744	97060	43100
	Frequency (SSR/Mb)	7.58	6.19	5.49	3.89	1.72
	Density (bp/Mb)	142.54	111.55	98.30	69.19	30.73
AAAG	Number of SSRs	2115	3419	7449	3011	695
	Total length (bp)	39336	60516	134672	52956	12220
	Frequency (SSR/Mb)	3.80	1.86	3.80	2.15	1.39
	Density (bp/Mb)	70.69	32.90	68.68	37.75	24.49
AATT	Number of SSRs	167	2166	1764	1007	279
	Total length (bp)	2844	38332	32752	17628	4864
	Frequency (SSR/Mb)	0.30	1.18	0.90	0.72	0.56
	Density (bp/Mb)	5.11	20.84	16.70	12.57	9.75
AAAAT	Number of SSRs	652	6646	3360	887	508
	Total length (bp)	14160	143850	70780	18960	10740
	Frequency (SSR/Mb)	1.17	3.61	1.71	0.63	1.02
	Density (bp/Mb)	25.45	78.22	36.10	13.52	21.52
AAAAG	Number of SSRs	846	1761	1228	1101	432
	Total length (bp)	18895	37900	26140	23915	9365
	Frequency (SSR/Mb)	1.52	0.96	0.63	0.79	0.87
	Density (bp/Mb)	33.95	20.61	13.33	17.05	18.77
AATAT	Number of SSRs	612	2662	946	889	761
	Total length (bp)	13630	61295	20375	19400	17180
	Frequency (SSR/Mb)	1.10	1.45	0.48	0.63	1.53
	Density (bp/Mb)	24.49	33.33	10.39	13.83	34.43
AAATT	Number of SSRs	35	6313	309	502	385
	Total length (bp)	725	138175	6600	11295	8790
	Frequency (SSR/Mb)	0.06	3.43	0.16	0.36	0.77
	Density (bp/Mb)	1.30	75.13	3.37	8.05	17.61
AGAGGG	Number of SSRs	189	78	107	198	7
	Total length (bp)	4836	1920	2736	5184	174
	Frequency (SSR/Mb)	0.34	0.04	0.06	0.14	0.01
	Density (bp/Mb)	8.69	1.04	1.40	3.70	0.35
AAAAAT	Number of SSRs	146	938	457	83	96
	Total length (bp)	3738	23424	11382	2064	2382
	Frequency (SSR/Mb)	0.26	0.51	0.23	0.06	0.19
	Density (bp/Mb)	6.72	12.74	5.81	1.47	4.77
ACATAT	Number of SSRs	87	360	421	303	426
	Total length (bp)	2490	9678	11988	8736	12360
	Frequency (SSR/Mb)	0.16	0.20	0.22	0.22	0.85
	Density (bp/Mb)	4.48	5.26	6.11	6.23	24.77
AAAAAG	Number of SSRs	109	239	209	120	67
	Total length (bp)	2790	5976	5232	3096	1704
	Frequency (SSR/Mb)	0.20	0.13	0.11	0.09	0.13
	Density (bp/Mb)	5.01	3.25	2.67	2.21	3.42

#### Dinucleotide repeats

**Fig. 4:**
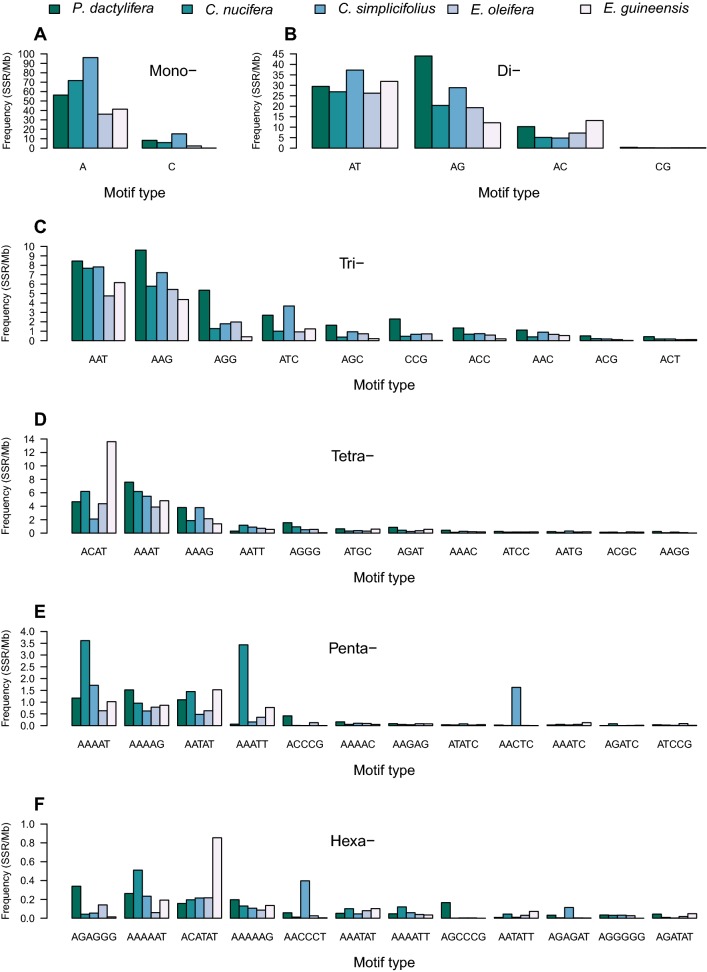
The most frequent SSR motif types in palm genomes. ABCDEF represent mono-, di-, tri-, tetra-, penta-, and hexanucleotide SSR types, respectively

The $(\hbox {AG})_{\mathrm{n}}$ motif type was the most predominant dinucleotide SSR in *P. dactylifera*, with a frequency of 44.02 SSR/Mb and occupying about 52.31% of all dinucleotide SSRs in this genome (Fig. 4b). The most frequent dinucleotide motif in *C. nucifera*, *C. simplicifolius*, *E. oleifera*, and *E. guineensis* was $(\hbox {AT})_{\mathrm{n}}$, with frequencies of 26.26–37.30 SSR/Mb and accounting for 51.20, 52.43, 49.54, and 55.58% of all dinucleotide SSRs in these genomes, respectively (Table 3). The $(\hbox {CG})_{\mathrm{n}}$ motif was the least frequent dinucleotide SSR (0.15–0.38 SSR/Mb) in all of the five genomes. Dinucleotide repeats ranged from 7–86, 7–31, 7–85, 7–118, and 7–41 repeats in length, respectively. The most predominant repeat numbers ranged between 7–28, 7–24, 7–42, 7–25, and 7–21, which accounted for 99.16, 99.93, 99.56, 99.38, and 97.65% of all dinucleotide SSRs, respectively (Fig. 3b).

#### Trinucleotide repeats

$(\hbox {AAG})_{\mathrm{n}}$ was the most frequent trinucleotide motif in *P. dactylifera* and *E. oleifera*, with frequencies of 9.61 and 5.44 SSR/Mb and accounting for 28.75 and 34.02% of all trinucleotide SSRs in these two genomes, respectively (Fig. 4c). The $(\hbox {AAT})_{\mathrm{n}}$ repeat was the most abundant motif in *C. nucifera*, *C. simplicifolius*, and *E. guineensis*, with frequencies of 7.69, 7.83, and 6.16 SSR/Mb and comprising about 42.57, 32.46, and 46.45% of all trinucleotide SSRs in these genomes, respectively. The $(\hbox {AGG})_{\mathrm{n}}$ and $(\hbox {ATC})_{\mathrm{n}}$ were also more frequent than other trinucleotide motifs, together accounting for 12.50–24.07% of all trinucleotide SSRs in the five palm genomes. $(\hbox {ACG})_{\mathrm{n}}$ and $(\hbox {ACT})_{\mathrm{n}}$ motifs were the least frequent trinucleotide SSRs in *P. dactylifera*, *C. nucifera*, *C. simplicifolius*, and *E. oleifera*, whereas $(\hbox {CCG})_{\mathrm{n}}$ and $(\hbox {ACG})_{\mathrm{n}}$ were the least abundant motifs in *E. guineensis*. Trinucleotide repeat counts ranged from 5–97, 5–21, 5–32, 5–59, and 5–42, respectively. Repeat numbers between 5–12, 5–20, 5–17, 5–12, and 5–9 accounted for 97.84, 99.94, 99.30, 97.45, and 91.84% of all trinucleotide SSRs in these genomes, respectively (Fig. 3c).

#### Tetranucleotide repeats

The $(\hbox {AAAT})_{\mathrm{n}}$ motif was the most abundant tetranucleotide repeat in *P. dactylifera* and *C. simplicifolius*, with frequencies of 7.58 and 5.49 SSR/Mb and occupying about 34.02 and 35.47% of all tetranucleotide SSRs in these two genomes, respectively (Table 3). The most frequent tetranucleotide motif in *E. oleifera* and *E. guineensis* was $(\hbox {ACAT})_{\mathrm{n}}$, accounting for 31.80 and 59.40% of all tetranucleotide SSRs in those genomes, respectively. $(\hbox {ACAT})_{\mathrm{n}}$ and $(\hbox {AAAT})_{\mathrm{n}}$ repeats were the predominant tetranucleotide motifs in *C. nucifera*, with almost identical frequencies of approximately 6.20 SSR/Mb, together comprising about 67.55% of all tetranucleotide SSRs in the genome (Fig. 4d). $(\hbox {AAAG})_{\mathrm{n}}$ and $(\hbox {AATT})_{\mathrm{n}}$ motifs were relatively frequent repeats in all five genomes. Tetranucleotide repeat counts ranged from 4–96, 4–15, 4–35, 4–31, and 4–33, respectively. Repeat numbers between 4–9, 4–12, 4–9, 4–9, and 4–10 accounted for 98.61, 99.60, 99.45, 99.01, and 98.62% of all tetranucleotide SSRs in these five genomes, respectively (Fig. 3d).

#### Pentanucleotide repeats

The $(\hbox {AAAAG})_{\mathrm{n}}$ motif was the most frequent pentanucleotide repeat in *P. dactylifera* and *E. oleifera*, followed by $(\hbox {AAAAT})_{\mathrm{n}}$ and $(\hbox {AATAT})_{\mathrm{n}}$ motifs. These three motif types together accounted for 67.01 and 60.42% of all pentanucleotide SSRs in those two genomes, respectively (Table 3). The most abundant motif in *C. nucifera* was $(\hbox {AAAAT})_{\mathrm{n}}$, which accounted for 35.54% of all pentanucleotide SSRs; meanwhile, $(\hbox {AATAT})_{\mathrm{n}}$ was the predominant pentanucleotide motif in *E. guineensis*, accounting for 31.68% of all pentanucleotide SSRs in that genome. The $(\hbox {AAAAT})_{\mathrm{n}}$ motif was also relatively frequent in *E. guineensis*, being the second most abundant type. $(\hbox {AAAAT})_{\mathrm{n}}$ and $(\hbox {AACTC})_{\mathrm{n}}$ motifs were the most abundant types in *C. simplicifolius* with similar frequencies of approximately 1.7 SSR/Mb, together accounting for 59.48% of the total number of pentanucleotide SSRs in this genome (Fig. 4e). Pentanucleotide repeat counts ranged from 4–73, 4–12, 4–10, 4–24, and 4–16, respectively. Repeat numbers between 4–6, 4–7, 4–6, 4–6, and 4–6 accounted for 97.90, 99.22, 99.23, 98.15, and 97.34% of all pentanucleotide SSRs in the five palm genomes, respectively (Fig. 3e).

#### Hexanucleotide repeats

Hexanucleotide motifs were found to have far lower frequency and density compared to other microsatellite repeat types. $(\hbox {AGAGGG})_{\mathrm{n}}$ and $(\hbox {AAAAAT})_{\mathrm{n}}$ motifs were the most abundant hexanucleotide types in *P. dactylifera*, together accounting for 25.36% of all hexanucleotide SSRs (Fig. 4f). The most abundant motifs in *C. nucifera* were $(\hbox {AAAAAT})_{\mathrm{n}}$ and $(\hbox {ACATAT})_{\mathrm{n}}$, together accounting for 39.76% of all hexanucleotide SSRs, while $(\hbox {AACCCT})_{\mathrm{n}}$ and $(\hbox {AAAAAT})_{\mathrm{n}}$ were the predominant hexanucleotide motifs in *C. simplicifolius*, together accounting for 28.07% of all hexanucleotide SSRs in the genome. The $(\hbox {ACATAT})_{\mathrm{n}}$ motif was found to be the most frequent type in *E. oleifera* and *E. guineensis*, with frequencies of below 0.86 SSR/Mb. Hexanucleotide repeat lengths ranged from 4–10, 4–11, 4–21, 4–17, and 4–20, respectively. Repeat numbers between 4–5, 4–5, 4–6, 4–5, and 4–5 accounted for 94.02, 96.45, 97.18, 91.68, and 90.08% of all hexanucleotide SSRs in the five palm genomes, respectively (Fig. 3f).

## Discussion

The availability of genomic sequences for several palm species provides the opportunity to elucidate and compare the distributions of microsatellites across these genomes. In a previous study, genomic microsatellite loci were screened for two Palmae species (*P. dactylifera* and *E. oleifera*) (Xiao et al. 2016). To the best of our knowledge, the present study is the first comprehensive report on the identification of microsatellites with 1–6 bp nucleotide motifs in five Palmae species: *P. dactylifera*, *C. nucifera*, *C. simplicifolius*, *E. oleifera*, and *E. guineensis*. Consistent search parameters were used to perform the same analysis for all five palm genomes. Computational approaches were utilized to elucidate and compare the relative frequency, relative density, and GC content of SSRs in these species. Perfect microsatellites were found to comprise 0.23–0.44% of the five palm genomes. The percentages of SSRs in species within the same genus (*E. oleifera* and *E. guineensis*) were comparable, and lower than in the other three palm genomes. This variation in the percentage of genome SSR content may arise from differences in computational methods used for SSR identification, the relative completeness of different genome assemblies as obviously observed in the genome assembly of *E. guineensis*, or real variation in microsatellite content among these species (Sharma et al. 2007).

The six types of SSRs were not equally represented in all five palm genomes. In general, mono- and dinucleotide repeats were found to prevail. More precisely, mononucleotide SSRs were the most frequent repeat type in *C. nucifera* and *C. simplicifolius*, consistent with previous findings in monocots and dicots (Sonah et al. 2011) and similar to what has been found for eukaryotic genomes overall (Sharma et al. 2007; Qi et al. 2015). Dinucleotide SSR repeats were the most abundant type in *P. dactylifera*, *E. oleifera*, and *E. guineensis*, which is consistent with prior findings for dicotyledons (Kumpatla and Mukhopadhyay 2005). Tri- and tetranucleotide SSR types were found to have very similar frequencies in the five palm genomes. Hexanucleotide repeats were the least frequent SSR type in all five species, which is similar to what has been seen in previous studies (Subramanian et al. 2002; Liu et al. 2017; Manee et al. 2019).

Previously, microsatellite abundances were found to be similar in species of the same genus (Shi et al. 2014). Here, only *E. oleifera* and *E. guineensis* are classified into the same genus, and these species did not have similar profiles overall. Interestingly, the overall frequency and density of SSRs were about the same in *P. dactylifera* and *C. simplicifolius*, suggesting potential similarity in the genomic structures of these two palm species. This is further supported by these genomes having similar abundances of SSRs by type, with the exception of mononucleotide SSRs.

Within each type of SSR, microsatellite motifs were found to vary greatly for each of the five palm genomes. Among mononucleotide repeats, the most abundant motif was $(\hbox {A/T})_{\mathrm{n}}$, accounting for 86.37–99.84% of the total number of mononucleotide SSRs. This observation is consistent with previous results from *Volvariella volvacea*, *Agaricus bisporus*, and *Coprinus cinereus* (Wang et al. 2014). Of dinucleotide SSRs, the $(\hbox {AT})_{\mathrm{n}}$ motif was the most frequent in all examined genomes except for *P. dactylifera*, and this trend was similar in dicots (Sonah et al. 2011), pineapple (Fang et al. 2016), cucumber (Cavagnaro et al. 2010), and sweet orange (Biswas et al. 2014). The most abundant dinucleotide repeat in *P. dactylifera* was $(\hbox {AG})_{\mathrm{n}}$, which is consistent with previous findings in *Brachypodium distachyon* (Sonah et al. 2011), wheat (Deng et al. 2016), and garden asparagus (Li et al. 2016). Among trinucleotide SSRs, the $(\hbox {AAT})_{\mathrm{n}}$ motif was the most predominant in *C. nucifera*, *C. simplicifolius*, and *E. guineensis*, and consistent with reports from garden asparagus (Li et al. 2016), cucumber (Cavagnaro et al. 2010), pineapple (Fang et al. 2016), and *Medicago truncatula* and *Populus trichocarpa* (Sonah et al. 2011). The $(\hbox {AAG})_{\mathrm{n}}$ was the dominant trinucleotide motif in *P. dactylifera* and *E. oleifera*, similar to previous reports in *Arabidopsis thaliana* (Sonah et al. 2011) and *Brassica* species (Shi et al. 2013). The AT-rich motifs (AAAT)$_{\mathrm{n}}$, (AAAG)$_{\mathrm{n}}$, $(\hbox {AATT})_{\mathrm{n}}$, $(\hbox {AAAAT})_{\mathrm{n}}$, $(\hbox {AAAAG})_{\mathrm{n}}$, $(\hbox {AATAT})_{\mathrm{n}}$, $(\hbox {AAATT})_{\mathrm{n}}$, $(\hbox {AAAAAT})_{\mathrm{n}}$, $(\hbox {ACATAT})_{\mathrm{n}}$, and $(\hbox {AAAAAG})_{\mathrm{n}}$ were the most abundant tetra-, penta- and hexanucleotide SSRs in the five palm genomes. Overall, the overrepresentation of $(\hbox {AT})_{\mathrm{n}}$ motifs in palm genomes can be explained by the fact that strand separation is easier for AT-rich than for GC-rich sequences, raising the possibility of slipped strand mispairing (Zhao et al. 2011). A previous study revealed that the $(\hbox {AAAT})_{\mathrm{n}}$, $(\hbox {AAAAT})_{\mathrm{n}}$, $(\hbox {AAAAT})_{\mathrm{n}}$, and $(\hbox {AAAAAT})_{\mathrm{n}}$ motifs also predominated in *Brassica* species (Shi et al. 2013).

GC content varies greatly among different genomes because of different selective constraints. It is important to identify the driving force behind GC content diversity in order to understand genome evolution across species. The overall GC content of eukaryotic genomes does not vary widely (Šmarda and Bureš 2012). However, in plants, grass genomes are known to have high GC content compared to other angiosperm families (Barow and Meister 2002; Šmarda and Bureš 2012). This study found the five palm genomes analyzed had lower GC content (28.12–39.65%) than do grasses (43.57–46.90%) (Singh et al. 2016), a number of Poaceae species (Deng et al. 2016), five monocots (43.57–46.14%), and two green algae (55.70 and 63.45%) (Zhao et al. 2014). In addition, GC content was not evenly distributed in three of the species, the exceptions being *C. nucifera* and *E. guineensis* ($\sim$ 32%). Variation in GC content within each SSR type was also observed across the five genomes, with the exception of tetranucleotide SSRs. Tri- and hexanucleotide SSRs were generally found to have the highest GC contents. The results also suggested that $(\hbox {A/T})_{\mathrm{n}}$ motifs are the most predominant in each genome, consistent with findings in previous reports (Sharma et al. 2007; Shi et al. 2013; Li et al. 2016). This can be interpreted as confirming high AT content in the majority of the analyzed SSRs.

SSRs make up a significant proportion of the eukaryotic genomes and are highly polymorphic, surpassing coding gene sequences in both respects (Katti et al. 2001). The high mutation rates of SSRs make them highly informative and useful for a wide range of applications such as evolutionary research, population genotyping, and marker-assisted breeding. Recent studies have utilized genome-wide approaches for the development of SSR markers in plants (Shi et al. 2014; Deng et al. 2016; Kumari et al. 2019). Perhaps the main advantage of this strategy is to produce a large number of SSR markers distributed evenly throughout the genome. The construction of a Palmae SSR database for the scientific community would evidently have a significant impact on genetic studies in those species.

Comparative analysis of SSRs in these five palm genomes will provide a better understanding of the nature of these important sequences and will facilitate research on the role of SSRs in genome organization. Such knowledge will serve many useful purposes, including, among many others, the isolation and development of abundant markers for genetic and evolutionary studies mentioned above. In particular, elucidating the most frequent repeats in palm genomes provides an essential starting point for the library-based selection of markers that will be informative in distinguishing populations and cultivars within a species, or even for cross-species applications. This further provides an important foundation for characterizing genetic diversity in palm germplasm and for performing selection on valuable or undesired attributes while also maintaining and/or improving diversity.

## Electronic supplementary material

Below is the link to the electronic supplementary material.

Supplementary material 1 (bed 19458 KB)

Supplementary material 2 (bed 13773 KB)

Supplementary material 3 (bed 2746 KB)

Supplementary material 4 (bed 6985 KB)

Supplementary material 5 (bed 4643 KB)
